# Survival analysis of additional radiotherapy after endoscopic therapy in patients with T1N0M0 esophageal cancer

**DOI:** 10.1097/MD.0000000000046017

**Published:** 2025-11-28

**Authors:** Minxian Zhuang, Xuefen Fang, Canmei Zhong, Tingxuan Huang, Fenglin Chen

**Affiliations:** a Department of Gastroenterology, Fujian Medical University Union Hospital, Fuzhou, Fujian Province, China.

**Keywords:** endoscopic therapy, esophageal cancer, radiotherapy, survival analysis

## Abstract

This study aims to investigate prognostic factors in patients with T1N0M0 esophageal cancer (EC) treated with endoscopic therapy (ET) and evaluate the impact of additional radiotherapy (RT) after ET. Data of patients with T1N0M0 EC undergoing ET were extracted from the Surveillance, Epidemiology, and End Results database (2004–2016). Patients were divided into ET and ET + RT groups. Propensity score matching was applied, and survival outcomes were assessed using Kaplan–Meier and Cox regression analyses, with subgroup analyses by T stage. After propensity score matching, 394 patients were included. Compared with ET alone, additional RT was associated with significantly worse overall survival and cancer-specific survival. Age, T stage, and RT were identified as independent prognostic factors. Subgroup analysis indicated that T1a patients did not benefit from RT, while T1b patients showed a slight trend toward improvement, but without statistical significance. Additional RT after ET does not improve survival in T1N0M0 EC, particularly in T1a patients, and may even be detrimental. These findings highlight the importance of individualized treatment and suggest that ET alone remains sufficient for most early-stage cases.

## 1. Introduction

Endoscopic resection (ER) has become the primary therapeutic approach for early-stage esophageal cancer (EC). According to the 2022 National Comprehensive Cancer Network guidelines,^[1]^ ER is the preferred treatment for localized T1a lesions (<2 cm, well or moderately differentiated), which are associated with a very low risk of lymph node or distant metastasis and excellent long-term survival after endoscopic therapy (ET).^[2]^ For tumors confined to the lamina propria, muscularis mucosae (T1a), or superficial submucosa (T1b), endoscopic submucosal dissection (ESD) or mucosal resection is feasible when preoperative staging excludes lymph node metastasis, vascular or lymphatic invasion, or poor differentiation.^[3,4]^

Nevertheless, ER alone is insufficient for certain high-risk patients. Lesions involving the muscularis mucosae or submucosa may still carry risks of recurrence and nodal metastasis.^[5,6]^ Therefore, additional treatment, such as surgery or chemoradiotherapy, is recommended in cases with positive vertical margins, lymphovascular invasion, submucosal invasion depth > 200 μm, or poorly differentiated carcinoma.^[7]^ The rationale for these strategies is to prevent recurrence and improve long-term survival.

Previous studies have suggested that ER followed by chemoradiotherapy can achieve outcomes comparable to surgery in T1b EC.^[8]^ However, the role of radiotherapy (RT) alone after ER has rarely been investigated. Most reports are small-scale, heterogeneous, or focus on chemoradiotherapy, leaving uncertainty about whether adjuvant RT provides additional survival benefit. Considering the generally favorable prognosis of early EC, the toxicity of RT may outweigh potential benefits and negatively affect quality of life.

Therefore, the present study aimed to evaluate the prognostic impact of additional RT after ER in patients with T1N0M0 EC. Using a large Surveillance, Epidemiology, and End Results (SEER)-based cohort with propensity score matching, we systematically assessed overall and cancer-specific survival (CSS). We hypothesized that adjuvant RT would not provide significant survival benefits compared with ER alone, particularly in T1a patients, and sought to clarify its potential role in T1b disease.

## 2. Materials and methods

### 2.1. Data source

This study is a retrospective cohort study based on the SEER database, which collects information on cancer incidence, mortality, and prevalence from selected US state and county populations (representing ~28% of the US population). We used SEER*Stat software (version 8.3.9.2) and the “SEER 18 Regs Custom Data (with additional treatment fields), Nov 2018 Sub (1975–2016 varying)” database for data extraction and analysis.

### 2.2. Study population

Patients pathologically diagnosed with T1N0M0 EC according to the 8th edition tumor node metastasis staging system jointly issued by the Union for International Cancer Control and the American Joint Committee on Cancer, who underwent ER between 2004 and 2016, were included. Patients meeting any of the following exclusion criteria were excluded: unclear pathology or tumor node metastasis stage, esophagogastric junction cancer, patients receiving RT before ET, patients receiving RT both before and after surgery, and those lacking treatment information, outcome events, or survival time.

Based on the above inclusion and exclusion criteria, a total of 1269 patients with T1N0M0 EC treated with ET alone or ET followed by additional RT were selected. The following data were extracted: demographic characteristics (age at diagnosis, race, sex), tumor-related information (tumor location, tumor size, depth of invasion, pathological type, histological grade, regional lymph node involvement, distant metastasis), treatment information (endoscopic treatment method, whether RT was received, whether chemotherapy was received), and follow-up data (survival time, survival status, cause of death).

### 2.3. Study endpoints

The primary endpoints of this study were overall survival (OS), defined as the time from diagnosis of EC to death from any cause, and CSS, defined as the time from diagnosis of EC to death specifically due to EC.

### 2.4. Statistical methods

Given the large number of cases in the SEER database and the potential for significant bias due to baseline differences, and considering the retrospective nature of this study, propensity score matching (PSM) was employed to balance baseline differences between groups. Statistical analyses were performed using IBM SPSS Statistics version 22.0 (IBM Corporation, Armonk). The chi-square test was used to compare baseline characteristics and tumor features between the ET and ET + RT groups. The R software environment, Statistics Essentials for R, and the SPSS PS Matching plug-in were installed to extend SPSS 22.0 with 1:n PSM functionality. Covariates included in the matching were: age, race, sex, tumor location, tumor size, T stage, pathological type, and histological grade. A caliper value of 0.2 and a matching ratio of 1:2 were applied for PSM. Univariate and multivariate survival analyses were performed using the Cox proportional hazards regression model to calculate hazard ratios and their 95% confidence intervals (CIs) to identify factors affecting the prognosis of T1N0M0 EC patients treated with ET. Subgroup analyses were performed based on relevant factors. Survival curves were plotted and survival rates were calculated using the Kaplan–Meier method. Survival analysis comparisons were performed using the log-rank test. A *P*-value < .05 was considered statistically significant.

## 3. Results

### 3.1. Patient characteristics

This study included 1269 patients with T1N0M0 EC diagnosed between 2004 and 2016, of whom 1133 (89.3%) received ET alone and 136 (10.7%) received ET followed by RT. After 1:2 PSM, 394 patients were matched (261 ET, 133 ET + RT). Baseline characteristics between the 2 groups were well balanced after matching, with no significant differences in age, race, sex, tumor location, tumor size, T stage, histological type, or grade (all *P* > .05) (Table 1). The median follow-up time was 49 months, and the median survival time of the entire matched cohort was 54 months. The 5-year OS and CSS rates were 46.7% and 71.9%, respectively.

**Table 1:** Baseline characteristics of patients before and after PSM.

	Before PSM				After PSM			
	Total	ET	ET + RT	*P*-value	Total	ET	ET + RT	*P*-value
	(n = 1269)	(n = 1133)	(n = 136)		(n = 394)	(n = 261)	(n = 133)	
Age (yr)				.201				.856
<65	362 (28.5)	332 (29.3)	30 (22.0)		84 (21.3)	54 (20.7)	30 (22.6)	
65–74	442 (34.8)	389 (34.3)	53 (39.0)		146 (37.1)	96 (36.8)	50 (37.6)	
≥75	465 (36.6)	412 (36.4)	53 (39.0)		164 (41.6)	111 (42.5)	53 (39.8)	
Race				.090				.982
Black	47 (3.7)	38 (3.4)	9 (6.6)		26 (6.6)	17 (6.5)	9 (6.8)	
White	1184 (93.3)	1063 (93.8)	121 (89.0)		354 (89.8)	235 (90.0)	119 (89.5)	
Other	38 (3.0)	32 (2.8)	6 (4.4)		14 (3.6)	9 (3.4)	5 (3.8)	
Sex				.992				.853
Male	1026 (80.9)	916 (80.8)	110 (80.9)		319 (81.0)	212 (81.2)	107 (80.5)	
Female	243 (10.1)	217 (19.2)	26 (19.1)		75 (19.0)	49 (18.8)	26 (19.5)	
Tumor location				.032				.609
Upper 1/3 esophagus	47 (3.7)	36 (3.2)	11 (8.1)		21 (5.3)	12 (4.6)	9 (6.8)	
Middle 1/3 esophagus	196 (15.4)	174 (15.4)	22 (16.2)		57 (14.5)	36 (13.8)	21 (15.8)	
Lower 1/3 esophagus	896 (70.6)	804 (71.0)	92 (67.6)		288 (73.1)	196 (75.1)	92 (69.2)	
Unknown	130 (10.2)	119 (10.5)	11 (8.1)		28 (7.1)	17 (6.5)	11 (8.3)	
Tumor size (mm)				<.001				.958
<10	274 (21.6)	255 (22.5)	19 (14.0)		54 (13.7)	35 (13.4)	19 (14.3)	
10–19	188 (14.8)	168 (14.8)	20 (14.7)		65 (16.5)	45 (17.2)	20 (15.0)	
20–29	83 (6.5)	67 (5.9)	16 (11.8)		48 (12.2)	32 (12.3)	16 (12.0)	
≥30	83 (6.5)	64 (5.6)	19 (14.0)		45 (11.4)	28 (10.7)	17 (12.8)	
Unknown	641 (50.5)	579 (51.1)	62 (45.6)		182 (46.2)	121 (46.4)	61 (45.9)	
T stage				<.001				.665
T1a	895 (70.5)	838 (74.0)	57 (41.9)		181 (45.9)	124 (47.5)	57 (42.9)	
T1b	206 (16.2)	160 (14.1)	46 (33.8)		124 (31.5)	79 (30.3)	45 (33.8)	
T1	168 (13.2)	135 (11.9)	33 (24.3)		89 (22.6)	58 (22.2)	31 (23.3)	
Histological type				<.001				.464
Squamous cell carcinoma	151 (11.9)	115 (10.2)	36 (26.5)		92 (23.4)	59 (22.6)	33 (24.8)	
Adenocarcinoma	1035 (81.6)	942 (83.1)	93 (68.4)		287 (72.8)	194 (74.3)	93 (69.9)	
Other	83 (6.5)	76 (6.7)	7 (5.1)		15 (3.8)	8 (3.1)	7 (5.3)	
Histological grade				<.001				.825
High/Middle differentiated	607 (47.8)	547 (48.3)	60 (44.1)		185 (47.0)	59 (22.6)	60 (45.1)	
Poorly/Undifferentiated	188 (14.8)	140 (12.4)	48 (35.3)		132 (33.5)	194 (74.3)	45 (33.8)	
Other	474 (37.4)	446 (39.4)	28 (20.6)		77 (19.5)	8 (3.1)	28 (21.1)	

ET = endoscopic therapy, PSM = propensity score matching, RT = radiotherapy.

### 3.2. Survival analysis

**Figure 1.**
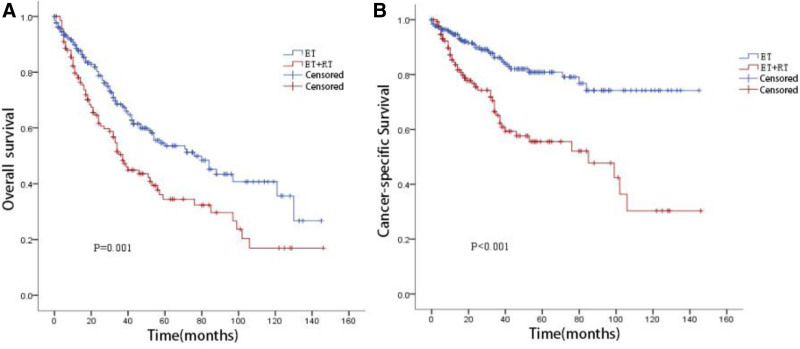
KM survival curves for patients in ET and ET + RT group after PSM. (A) KM overall survival curves for patients. (B) KM cancer specific survival curves for patients. ET = endoscopic therapy, KM = Kaplan–Meier, PSM = propensity score matching, RT = radiotherapy.

Kaplan–Meier survival analysis was performed for the ET and ET + RT groups after PSM (Fig. 1). The median survival time was 76 months (95% CI: 52.6–99.3 months) for the ET group and 37 months (95% CI: 30.6–43.4 months) for the ET + RT group. The 5-year OS rates were 53.6% and 34.5%, and the 5-year CSS rates were 80.9% and 55.6%, respectively. Patients in the ET group had significantly better OS (log-rank *P* = .001) and CSS (log-rank *P* < .001) compared with those in the ET + RT group.

### 3.3. Cox regression analysis

Univariate analysis (Tables 2 and 3) showed that age (*P* < .001), T stage (*P* = .005), and RT (*P* = .001) significantly affected OS. Age (*P* = .002), T stage (*P* = .008), pathological type (*P* = .027), and RT (*P* < .001) significantly affected CSS.

**Table 2:** Cox univariate and multivariate analysis of overall survival after PSM.

	Univariate analysis	*P*-value	Multivariate analysis	*P*-value
	HR (95% CI)		HR (95% CI)	
Age (yr)		<.001		<.001
<65	—		—	
65–74	1.588 (0.946–2.665)	.080	1.689 (1.004–2.840)	.048
≥75	3.350 (2.071–5.418)	<.001	3.515 (2.171–5.691)	<.001
Race		.374	—	
Black	—			
White	0.739 (0.447–1.222)	.239		
Other	0.485 (0.142–1.657)	.248		
Sex			—	
Male	—			
Female	0.948 (0.656–1.372)	.778		
Tumor location		.190	—	
Upper 1/3 esophagus	—			
Middle 1/3 esophagus	2.465 (0.959–6.333)	.061		
Lower 1/3 esophagus	1.818 (0.742–4.454)	.191		
Tumor size (mm)		.140	—	
<10	—			
10–19	1.285 (0.664–2.485)	.456		
20–29	1.957 (1.011–3.786)	.046		
≥30	1.454 (0.709–2.980)	.307		
T stage				
T1a	—		—	
T1b	1.565 (1.148–2.134)	.005	1.411 (1.032–1.930)	.031
Histological type		.211		
Squamous cell carcinoma	—			
Adenocarcinoma	0.811 (0.573–1.148)	.237		
Other	1.357 (0.682–2.700)	.385		
Histological grade				
High/Middle differentiated	—			
Poorly/Undifferentiated	1.034 (0.741–1.444)	.842		
Radiotherapy				
No	—		—	
Yes	1.690 (1.249–2.288)	.001	1.744 (1.286–2.366)	<.001

CI = confidence interval, HR = hazard ratio, PSM = propensity score matching.

**Table 3:** Cox univariate and multivariate analysis of cancer-specific survival after PSM.

	Univariate analysis	*P*-value	Multivariate analysis	*P*-value
	HR (95% CI)		HR (95% CI)	
Age (yr)		.002		<.001
<65	—		—	
65–74	1.298 (0.652–2.584)	.458	1.705 (0.827–3.515)	.149
≥75	2.502 (1.324–4.726)	.005	3.295 (1.690–6.426)	<.001
Race		.374	—	
Black	—			
White	0.583 (0.300–1.133)	.111		
Other	0.838 (0.230–3.056)	.789		
Sex			—	
Male	—			
Female	0.936 (0.548–1.598)	.807		
Tumor location		.326	—	
Upper 1/3 esophagus	—			
Middle 1/3 esophagus	2.135 (0.625–7.294)	.226		
Lower 1/3 esophagus	1.375 (0.429–4.407)	.592		
Tumor size (mm)		.072	—	
<10	—			
10–19	1.376 (0.461–4.109)	.567		
20–29	2.734 (0.963–7.765)	.059		
≥30	2.018 (0.660–6.170)	.218		
T stage				
T1a	—		—	
T1b	1.852 (1.174–2.919)	.008	1.593 (1.001–2.535)	.045
Histological type		.027		.111
Squamous cell carcinoma	—		—	
Adenocarcinoma	0.621 (0.384–1.004)	.052	0.770 (0.469–1.265)	.302
Other	1.659 (0.717–3.840)	.237	1.817 (0.762–4.331)	.178
Histological grade			—	
High/Middle differentiated	—			
Poorly/Undifferentiated	1.167 (0.727–1.874)	.523		
Radiotherapy				
No	—		—	
Yes	2.790 (1.807–4.309)	<.001	2.650 (1.702–4.126)	<.001

CI = confidence interval, HR = hazard ratio, PSM = propensity score matching.

Multivariate analysis incorporating these risk factors showed that age (*P* < .001), T stage (*P* = .031), and RT (*P* < .001) were independent predictors of OS. The risk of death for T1b patients was 1.411 times that of T1a patients (95% CI: 1.032–1.930, *P* = .031). The risk of death for patients receiving additional RT after ET was 1.744 times that of patients not receiving additional RT (95% CI: 1.286–2.366, *P* < .001). Age (*P* < .001), T stage (*P* = .045), and RT (*P* < .001) were also independent predictors of CSS. The risk of cancer-specific death for T1b patients was 1.593 times that of T1a patients (95% CI: 1.001–2.535, *P* = .045). The risk of cancer-specific death for patients receiving additional RT after ET was 2.650 times that of patients not receiving additional RT (95% CI: 1.702–4.126, *P* < .001).

### 3.4. Subgroup survival analysis

#### 3.4.1. Age subgroup analysis

##### 3.4.1.1. <65 years subgroup (Fig. 2A and B)

Due to a low number of events, 1- and 3-year OS/CSS were calculated. The ET group had 1-year OS 92.5%, 3-year OS 79.4%, 1-year CSS 94.2%, and 3-year CSS 91.2%. The ET + RT group had 1-year OS 89.4%, 3-year OS 66.0%, 1-year CSS 92.8%, and 3-year CSS 68.6%. The ET group had significantly better CSS (log-rank *P* = .043), while OS did not differ significantly between groups (log-rank *P* = .099).

**Figure 2.**
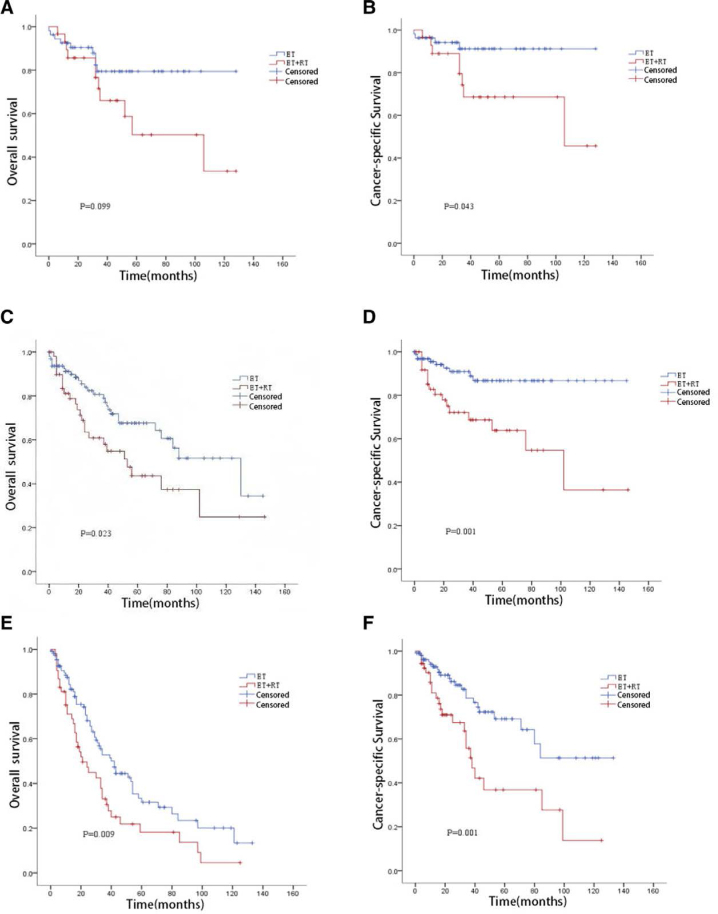
KM survival curves for patients in age subgroups. (A) KM overall survival curves for <65-year-old patients; (B) KM-cancer–specific survival curves for <65-year-old patients; (C) KM overall survival curves for 65- to 74-year-old patients; (D) KM-cancer–specific survival curves for 65- to 74-year-old patients; (E) KM overall survival curves for ≥75-year-old patients; (F) KM cancer specific survival curves for ≥75-year-old patients. KM = Kaplan–Meier.

##### 3.4.1.2. 65 to 74 years subgroup (Fig. 2C and D)

Median survival time was 88 months for ET versus 53 months for ET + RT. The 5-year OS was 67.6% versus 43.6%, and the 5-year CSS was 86.7% versus 63.8%. The ET group had significantly better OS (log-rank *P* = .023) and CSS (log-rank *P* = .001).

##### 3.4.1.3. ≥75 years subgroup (Fig. 2E and F)

Median survival time was 42 months for ET versus 21 months for ET + RT. The 5-year OS was 31.6% versus 18.2%, and the 5-year CSS was 69.1% versus 36.8%. The ET group had significantly better OS (log-rank *P* = .009) and CSS (log-rank *P* = .001).

#### 3.4.2. T stage subgroup analysis

##### 3.4.2.1. T1a stage subgroup (Fig. 3A and B)

Median survival time was 121 months for ET versus 32 months for ET + RT. The 5-year OS was 65.1% versus 31.1%, and the 5-year CSS was 90.4% versus 58.5%. The ET group had significantly better OS (log-rank *P* < .001) and CSS (log-rank *P* < .001).

**Figure 3.**
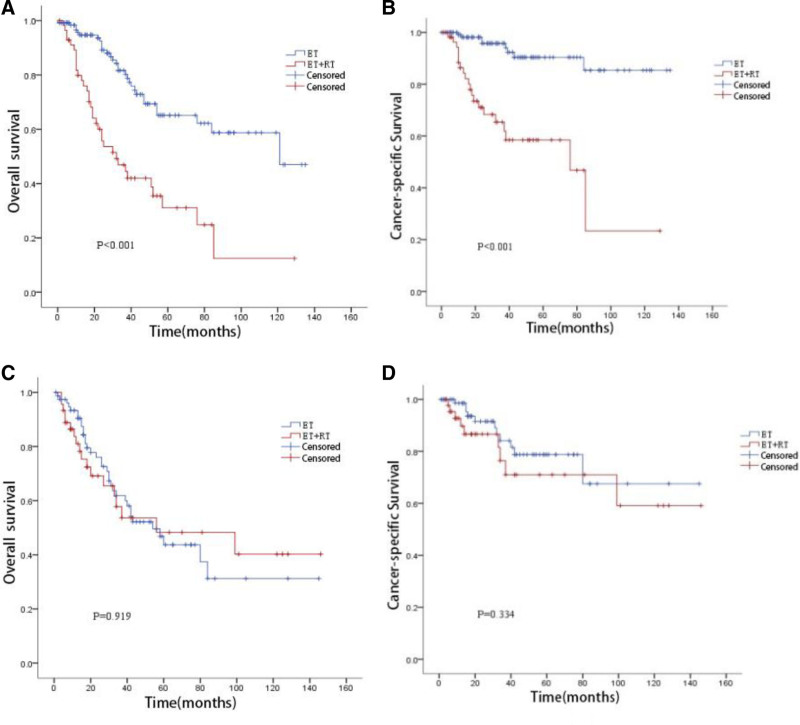
T stage subgroup analysis.

##### 3.4.2.2. T1b stage subgroup (Fig. 3C and D)

Median survival time was 54 months for ET versus 56 months for ET + RT. The 5-year OS was 43.7% versus 48.3%, and the 5-year CSS was 78.8% versus 71.0%. There were no significant differences in OS (log-rank *P* = .919) or CSS (log-rank *P* = .334) between the ET and ET + RT groups.

## 4. Discussion

This study demonstrated that in patients with T1N0M0 EC, ET alone achieved better OS and CSS than ET combined with RT. Notably, additional RT was associated with worse outcomes in T1a patients, while in T1b patients, it showed only a nonsignificant trend toward improved survival. These findings suggest that routine use of RT after ET may not be justified, particularly in low-risk T1a disease.

For early EC, previous studies have confirmed that ET yields outcomes comparable to radical surgery.^[9–12]^ However, the impact of additional RT after ET has been less well studied. Our SEER-based retrospective cohort with PSM addressed this gap and identified age, T stage, and RT as independent prognostic factors. Stratified analyses showed poorer prognosis in elderly and T1b patients but clear harm from RT in T1a disease.

Our results align with prior investigations. Hisano et al^[13]^ found that additional RT after ESD improved locoregional control but did not significantly affect 3-year OS or CSS. Similarly, Ikeda et al^[14]^ reported no significant difference in recurrence-free survival between patients receiving additional chemoradiotherapy and those under observation. These studies, together with our large population-based analysis, suggest that while RT may enhance local control, it does not confer a survival advantage.

The discrepancy between local control and survival may relate to comorbidities and treatment toxicity. Several studies reported that for pT1a-muscularis mucosae ESCC without vascular invasion, the lymph node metastasis rate was low (≈5%–6%) regardless of additional treatment, whereas with vascular invasion, the risk increased to ~20%.^[15–19]^ Meanwhile, adverse events from chemoradiotherapy were not negligible (≈2.6%), including radiation pneumonitis, myocardial infarction, and thromboembolism, with a treatment-related mortality of 1.3%.^[20–22]^ Such complications may offset potential survival gains, especially in T1a patients at low metastatic risk.

Histological subtype further influences outcomes. The majority of our patients had esophageal adenocarcinoma (EAC), in which lymph node metastasis rates are very low for T1a without lymphovascular invasion (0%–0.5%).^[23]^ For T1b with invasion ≤500 μm, the risk is 11% to 17%, and for deeper invasion, it can reach 8.6% to 36.6%. Long-term survival after ET in pT1a EAC is excellent, with 5-year OS rates of 91% to 100% and CSS rates of 96% to 100%,^[24,25]^ comparable or superior to surgery. Thus, the 2020 JGES guidelines recommend no additional treatment after R0 resection in well-differentiated T1a EAC without lymphovascular invasion.^[26,27]^ Moreover, Shapiro et al^[28]^ reported that EAC is less sensitive to chemoradiotherapy than squamous cell carcinoma, which may partly explain the poor outcomes in the ET + RT group.

### 4.1. Limitations and clinical implications

The use of the SEER database has inherent limitations. It does not provide detailed information on RT plans (dose, field, fractionation), ER techniques (e.g., ESD vs endoscopic mucosal resection), use of concurrent chemotherapy, or patient comorbidities and nutritional status, all of which may significantly influence treatment outcomes. Moreover, SEER lacks data on recurrence, disease-free survival, and treatment-related complications such as radiation esophagitis, strictures, pneumonitis, or cardiac injury, which are critical for evaluating the true impact of additional RT. In addition, as a retrospective cohort study, residual confounding remains possible despite the use of PSM to balance baseline characteristics.

Nevertheless, by analyzing a large population-based cohort with rigorous statistical adjustment, our study provides the most comprehensive evidence to date specifically evaluating RT alone after ET in early EC. Clinically, these findings indicate that routine RT after ET is not beneficial for T1a patients and should be considered with caution in T1b patients. They also underscore the importance of individualized treatment planning and highlight the need for future large-scale prospective studies with detailed treatment and complication data to validate these results and define precise indications for additional RT in early EC.

## 5. Conclusion

Age, T stage, and the use of additional RT are independent prognostic factors for patients with T1N0M0 EC treated with ET. Importantly, our findings clearly indicate that RT after ET is not recommended for T1a patients, as it is associated with worse survival outcomes. For T1b patients, additional RT did not confer a significant survival advantage, and its role remains uncertain. Therefore, treatment decisions should prioritize endoscopic therapy alone for T1a disease, while the optimal indications for additional RT in T1b disease warrant further investigation through prospective studies.

## Author contributions

**Conceptualization:** Minxian Zhuang, Xuefen Fang, Canmei Zhong, Tingxuan Huang, Fenglin Chen.

**Data curation:** Minxian Zhuang, Xuefen Fang, Tingxuan Huang, Fenglin Chen.

**Formal analysis:** Xuefen Fang, Fenglin Chen.

**Investigation:** Minxian Zhuang, Xuefen Fang, Canmei Zhong, Fenglin Chen.

**Methodology:** Minxian Zhuang, Xuefen Fang, Canmei Zhong, Tingxuan Huang, Fenglin Chen.

**Supervision:** Minxian Zhuang, Xuefen Fang, Canmei Zhong, Fenglin Chen.

**Validation:** Minxian Zhuang, Xuefen Fang, Canmei Zhong, Tingxuan Huang, Fenglin Chen.

**Visualization:** Minxian Zhuang, Xuefen Fang, Canmei Zhong, Tingxuan Huang, Fenglin Chen.

**Writing – original draft:** Minxian Zhuang, Fenglin Chen.

**Writing – review & editing:** Minxian Zhuang, Fenglin Chen.
